# Hospital market concentration and hospital adoption of artificial intelligence and machine learning

**DOI:** 10.1093/haschl/qxag287

**Published:** 2026-10-16

**Authors:** Jie Chen, Alice Yan

**Affiliations:** Department of Health Policy and Management, School of Public Health, University of Maryland at College Park, College Park, MD 20742, USA; Hospital And Public health interdisciPlinarY research (HAPPY) Lab, University of Maryland at College Park, College Park, MD 20742, USA; Center on Aging, University of Maryland at College Park, College Park, MD 20742, USA; Center for Seniors Uniting Nationwide to Support Health, INtegrated care, and Economics (SUNSHINE), University of Maryland at College Park, College Park, MD 20742, USA; Department of Health Policy and Management, School of Public Health, University of Maryland at College Park, College Park, MD 20742, USA; Hospital And Public health interdisciPlinarY research (HAPPY) Lab, University of Maryland at College Park, College Park, MD 20742, USA; Center on Aging, University of Maryland at College Park, College Park, MD 20742, USA

**Keywords:** hospital market concentration, artificial intelligence, machine learning, health systems, accountable care organizations

## Abstract

**Introduction:**

We examined associations of hospital market concentration and organizational characteristics with artificial intelligence (AI) and machine learning (ML) use.

**Methods:**

We analyzed 2025 hospital survey data, measuring concentration with the Herfindahl–Hirschman index (HHI). Primary outcomes measured current use of AI/ML both integrated into and outside electronic health records (EHRs), and function counts requiring tools from both the EHR developer and another source. Alternative measures allowed either setting or source. Standard errors were clustered by hospital referral region.

**Results:**

High vs low concentration was associated with a 6.7-percentage-point higher adjusted probability of generative-AI use in both settings (95% CI, 0.2-13.2), but not with any use (0.2; 95% CI, −4.9 to 5.2). Moderate concentration was associated with 0.20 fewer generative functions requiring both sources (95% CI, −0.39 to −0.01). System affiliation and accountable care organization (ACO) participation were positively associated with all 5 outcomes under both definitions.

**Conclusions:**

Market concentration was not consistently associated with AI/ML use across technologies and outcome definitions. System affiliation and ACO participation were consistently associated with greater use.

Key points

- Associations between market concentration and AI/ML use were limited and varied across outcome definitions.
- System affiliation and accountable care organization participation were consistently associated with greater AI/ML use and more functions.
- Rural hospitals had lower rates of any non–ML predictive use and fewer generative functions using any source.

## Introduction

Hospitals are using machine learning (ML) predictive models, other predictive analytics, and generative artificial intelligence (AI) for clinical and administrative functions.^1-4^ Implementation requires data infrastructure, technical expertise, governance, workflow changes, and funding.^5-8^ Differences in these resources may shape which hospitals use AI and which functions they support. Understanding these differences can help identify hospitals needing implementation support.

Market competition and organizational resources may influence technology use in different ways. As hospital markets consolidate,^9-11^ changes in prices, services, and quality raise concerns about access and equity.^12-14^ Competition may encourage technology investment as hospitals seek efficiency or attract patients.^15,16^ Studies of MRI, electronic medical records, and cardiac catheterization illustrate these incentives.^17-19^ Hospitals with greater scale or shared resources may also be better able to finance infrastructure and ongoing oversight.^5,7^ Consolidation alone does not ensure effective technology integration.^20^

Hospital concentration is distinct from health-system affiliation, which may provide shared infrastructure,^21^ and accountable care organization (ACO) participation, which may encourage prediction and care coordination. Unequal access to these resources could disadvantage independent and rural hospitals.^22,23^ Everson and colleagues reported organizational differences in current or planned generative-AI use integrated into electronic health records (EHRs).^24^ Health information exchange studies also show the relevance of hospital and EHR vendor markets.^25,26^ Less is known about current predictive and generative use across EHR settings and the functions supported.

We examined whether hospital-market concentration was associated with adoption and breadth of AI and ML use after accounting for hospital organizational characteristics and community context. We hypothesized that greater concentration would be associated with lower adoption because of reduced competitive pressure, while system affiliation and ACO participation would be associated with greater adoption.

## Data and methods

We linked the 2025 American Hospital Association (AHA) Information Technology Supplement, 2024 AHA Annual Survey, and 2025 Area Health Resources File.^27-29^ The IT survey provided hospital-reported technology use; the other sources supplied hospital and county characteristics. The study included US general medical and surgical hospitals, with hospitals as the unit of analysis. Institutional review board approval and informed consent were not required because the data contained no identifiable individual-level information.

Three primary binary outcomes measured current use of ML-based predictive models, non–ML-based predictive models, and generative AI. Survey questions distinguished tools integrated into the EHR from tools not integrated into the EHR. For each technology, use in both settings was coded 1; use in only one or neither was coded 0. Two additional outcomes counted predictive functions (0-7) and generative functions (0-8). The function questions distinguished tools from the EHR developer from tools from another source, including third-party or self-developed tools. In the primary count, a function contributed 1 only if both source categories supported that same function; otherwise, it contributed 0. Each function counted at most once. EHR integration and tool source are distinct measures (Supplement 1). Outcome-specific sample sizes are reported in Table S2B.

We calculated the Herfindahl–Hirschman Index (HHI) within hospital referral regions (HRRs).^30^ Each hospital's share was its inpatient admissions divided by total admissions across hospitals in the same HRR. HHI was the sum of squared shares multiplied by 10 000. Commonly owned hospitals remained separate facilities. Study categories were low (<1000), moderate (1000-1800), and high (>1800) concentration.

We used logistic regressions for binary outcomes and Poisson regressions for counts. Average marginal effects and 95% confidence intervals (CIs) are reported as percentage-point differences or differences in function counts. Robust standard errors were clustered by HRR. Clustering accounts for similarities among hospitals in the same market, which share the same HHI. Unadjusted models included HHI alone. Adjusted models included system affiliation, ACO participation, rurality, ownership, size, teaching status, staffing, payer mix, and county characteristics. To retain hospitals with missing ACO responses, we coded ACO participation, nonparticipation, and nonresponse separately. Unadjusted and adjusted models used the same outcome-specific samples.

In sensitivity analyses, we used alternative outcome definitions. Binary outcomes were coded 1 for current use in either or both EHR settings and 0 for neither. Each function counted once if supported by either or both tool sources (Supplement 3). Primary and alternative models used identical outcome-specific samples, covariates, and HRR clustering. Analyses were performed in Stata/MP 18. Confidence intervals were not adjusted for multiple comparisons.

## Results

In the study sample, use in both EHR settings was reported by 52.0% of hospitals for ML-based predictive models (*N* = 1959), 45.9% for non–ML predictive models (*N* = 1817), and 61.1% for generative AI (*N* = 2017). Generative-AI use was EHR-only in 8.5%, outside-EHR-only in 10.5%, and absent in 19.9%; 80.1% reported any use. Mean counts using both sources were 1.21 predictive and 0.54 generative functions (*N* = 1872). Missing covariates other than ACO responses excluded 12-13 hospitals per outcome. Regression samples were 1947, 1804, and 2004 for the 3 binary outcomes and 1859 for each count, retaining over 99% of each descriptive outcome sample (Table S2B).

Figure 1 maps hospital use in both EHR integration settings for ML-based predictive models (Panel A), non–ML-based predictive models (Panel B), and generative AI (Panel C) across HRR market concentration categories. Hospitals with and without use in both settings were present at every concentration level, often within the same HRR. Hospital locations were more clustered along the East and West Coasts and around the Great Lakes, and more dispersed across the Mountain West and Great Plains. Each marker represents a hospital, so more markers may simply reflect more hospitals in an area. The maps do not show regional use rates or adjusted associations.

**Figure 1.**
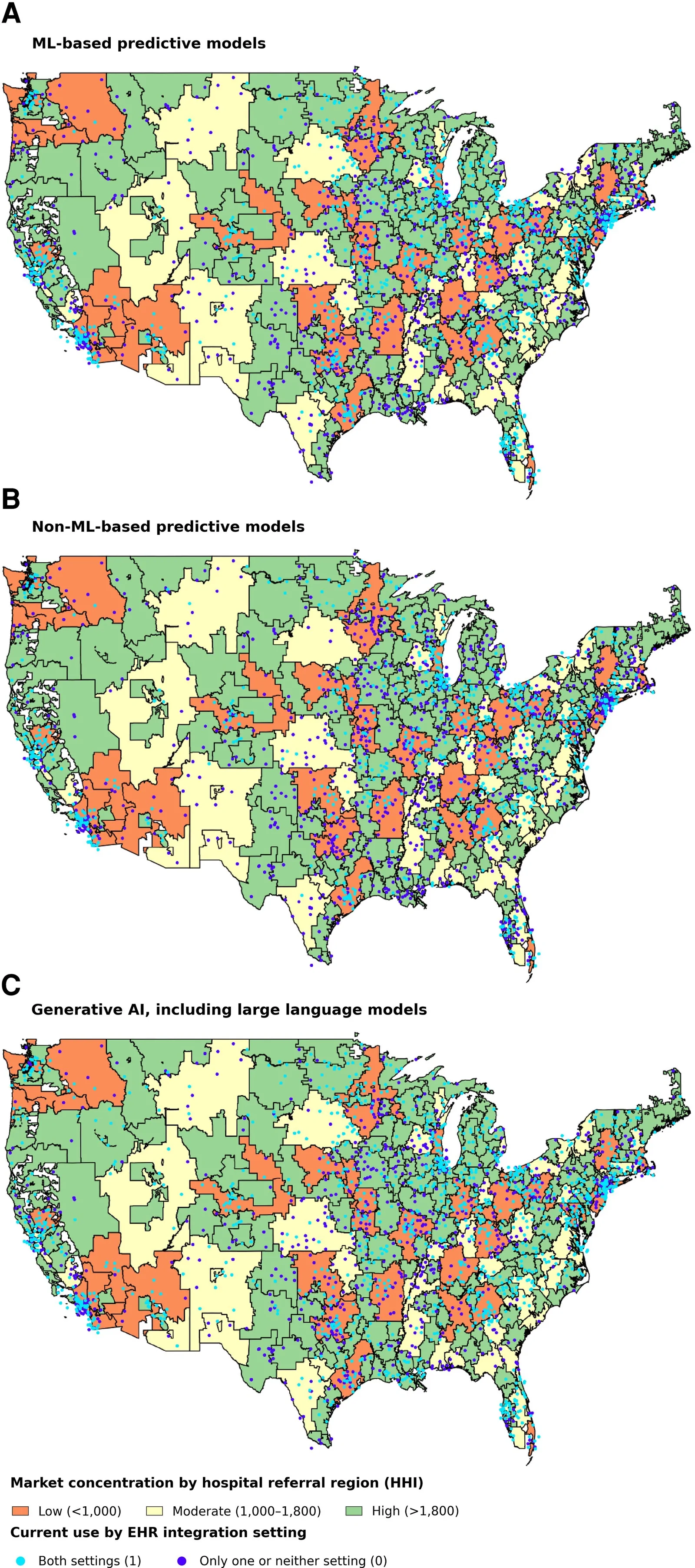
Hospital AI/ML use and market concentration. (Panel A) ML-based predictive models. (Panel B) Non–ML-based predictive models. (Panel C) Generative AI, including large language models. Hospital markers: light-blue = current use in both EHR integration settings (code 1); purple = use in only one or neither setting (code 0). Both settings means integrated into and not integrated into the EHR. Planned use is not current use. Hospitals with “do not know” or missing responses in either setting are excluded. HRR shading: orange = low HHI (<1000); yellow = moderate HHI (1000-1800); green = high HHI (>1800). HHI uses squared facility-level inpatient-admission shares within each HRR, multiplied by 10 000; commonly owned hospitals remain separate facilities. Sources: 2025 AHA IT Supplement and 2024 AHA Annual Survey. These maps show hospital locations and HRR concentration, not regional use rates or adjusted associations. Marker density partly reflects the number of hospitals.

In unadjusted models, high vs low concentration was associated with lower non–ML predictive-model use in both settings (−9.9% points; 95% CI, −19.5 to −0.3) and fewer generative functions using both sources (−0.195; 95% CI, −0.388 to −0.003). The ML predictive-model association was not statistically significant (Table 1).

**Table 1.** Full regression results for AI/ML use in both EHR integration settings and function counts using both tool sources.

Covariate/contrast	ML predictive use (both settings) *N* = 1947	Non–ML predictive use (both settings) *N* = 1804	Generative-AI use (both settings) *N* = 2004	Predictive functions (both sources) *N* = 1859	Generative functions (both sources) *N* = 1859
**Model 1: Unadjusted**					
Moderate vs low HHI	−8.2 (−19.5, 3.0)	−8.0 (−19.6, 3.6)	−5.0 (−15.5, 5.6)	−0.125 (−0.509, 0.260)	−0.261 (−0.462, −0.061)
High vs low HHI	−7.8 (−16.3, 0.8)	−9.9 (−19.5, −0.3)	1.7 (−5.9, 9.2)	−0.028 (−0.359, 0.304)	−0.195 (−0.388, −0.003)
**Model 2: Adjusted**					
Moderate vs low HHI	−0.2 (−9.9, 9.5)	−1.2 (−10.7, 8.4)	0.5 (−7.9, 8.8)	0.034 (−0.329, 0.397)	−0.202 (−0.391, −0.014)
High vs low HHI	−0.1 (−7.6, 7.4)	−3.3 (−11.7, 5.1)	6.7 (0.2, 13.2)	0.159 (−0.156, 0.475)	−0.111 (−0.290, 0.069)
System affiliated vs independent	39.5 (31.9, 47.2)	38.2 (31.5, 44.9)	30.9 (23.1, 38.7)	0.973 (0.749, 1.197)	0.300 (0.140, 0.460)
ACO participant vs nonparticipant	21.7 (14.0, 29.3)	18.2 (9.9, 26.5)	20.2 (13.2, 27.2)	0.459 (0.172, 0.746)	0.214 (0.026, 0.402)
ACO nonresponse vs nonparticipant	−1.4 (−11.5, 8.8)	−0.5 (−10.3, 9.4)	5.2 (−3.6, 14.0)	0.045 (−0.252, 0.342)	0.107 (−0.050, 0.263)
Rural vs urban	−3.5 (−11.2, 4.2)	0.7 (−6.7, 8.0)	−4.1 (−11.7, 3.5)	0.071 (−0.275, 0.418)	−0.020 (−0.213, 0.174)
Nongovernment vs government	16.3 (7.9, 24.7)	18.1 (8.1, 28.2)	18.4 (10.3, 26.5)	0.397 (0.021, 0.773)	0.191 (0.013, 0.370)
<50 vs 50-99 beds	4.1 (−3.2, 11.3)	6.2 (−1.2, 13.5)	4.9 (−2.6, 12.4)	0.303 (−0.047, 0.653)	0.183 (−0.054, 0.420)
≥100 vs 50-99 beds	12.6 (5.3, 19.9)	11.9 (4.3, 19.6)	7.9 (0.7, 15.0)	0.242 (−0.027, 0.511)	−0.005 (−0.167, 0.158)
Teaching vs nonteaching	−6.2 (−10.9, −1.5)	−5.0 (−10.6, 0.6)	−2.1 (−7.4, 3.1)	−0.258 (−0.453, −0.064)	−0.095 (−0.204, 0.013)
RN staffing >75th percentile	−0.2 (−6.2, 5.8)	1.2 (−4.8, 7.1)	−0.8 (−6.5, 5.0)	−0.098 (−0.335, 0.138)	−0.116 (−0.242, 0.010)
Medicare discharge rate >75th percentile	−1.6 (−7.3, 4.0)	−4.2 (−10.1, 1.7)	−3.8 (−9.1, 1.4)	−0.015 (−0.236, 0.206)	−0.017 (−0.154, 0.119)
Medicaid discharge rate >75th percentile	−0.9 (−6.8, 5.1)	2.7 (−3.7, 9.1)	1.6 (−3.6, 6.7)	0.207 (−0.035, 0.449)	0.083 (−0.075, 0.241)
County FQHC supply >75th percentile	1.6 (−4.6, 7.8)	3.2 (−3.2, 9.5)	−3.0 (−8.4, 2.4)	0.145 (−0.103, 0.393)	0.069 (−0.057, 0.194)
County minority-race share >75th percentile	−1.4 (−7.5, 4.7)	−0.7 (−7.8, 6.4)	0.6 (−4.9, 6.1)	0.152 (−0.092, 0.395)	0.107 (−0.056, 0.270)
County age ≥65 share >75th percentile	2.8 (−3.8, 9.3)	−2.9 (−10.3, 4.5)	5.4 (−0.5, 11.3)	−0.100 (−0.361, 0.161)	−0.005 (−0.166, 0.157)
County median income >75th percentile	7.4 (0.8, 14.1)	5.0 (−1.8, 11.7)	3.4 (−2.4, 9.3)	0.193 (−0.023, 0.409)	0.127 (−0.005, 0.258)

Values are average marginal effects (95% CIs). Binary outcomes are percentage-point differences; count outcomes are differences in numbers of functions. Model 1 includes HHI only; Model 2 includes all covariates shown. Logistic regressions were used for binary outcomes and Poisson regressions for counts. Standard errors are clustered by HRR. Both models use identical outcome-specific samples. Confidence intervals are not adjusted for multiple comparisons.

Reference categories are shown in each contrast. For percentile-based covariates, the reference is the 75th percentile or below. RN staffing is full-time-equivalent registered nurses per 1000 inpatient days. FQHC denotes federally qualified health center; county racial composition is the share identifying as a minority race. ACO nonresponse is a separate category. HHI: low <1000; moderate 1000-1800; high >1800.

Primary binary outcomes equal 1 for current use both integrated into and not integrated into the EHR, and 0 for current use in only one setting or neither setting, among hospitals with classifiable responses. For each function, the primary count adds 1 when the hospital reports using tools from both its EHR developer and another source; ranges are 0-7 predictive and 0-8 generative functions.

AI, artificial intelligence; ML, machine learning; ACO, accountable care organization; EHR, electronic health record; HRR, hospital referral region; HHI, Herfindahl–Hirschman index.

After adjustment, high concentration was associated with a 6.7-percentage-point higher probability of generative-AI use in both settings (95% CI, 0.2-13.2). Its association with the generative function count was not significant (−0.11; 95% CI, −0.29 to 0.07). Moderate concentration was associated with 0.20 fewer generative functions using both sources (95% CI, −0.39 to −0.01). Neither concentration category was significantly associated with predictive-model use or predictive function counts.

System affiliation was associated with 30.9-39.5-percentage-point higher probabilities of use of the 3 technologies in both settings, 0.97 additional predictive functions and 0.30 additional generative functions. Corresponding ACO associations were 18.2%-21.7% points, 0.46 functions, and 0.21 functions. Rural–urban differences were not significant for any primary outcome.

Under the alternative definitions, neither concentration category was significantly associated with any adjusted outcome. The high-concentration estimate for any generative-AI use was 0.2% points (95% CI, −4.9 to 5.2). System affiliation and ACO participation remained positively associated with all 5 outcomes. Rural hospitals had a lower probability of any non–ML predictive use (−6.8% points; 95% CI, −12.2 to −1.4) and fewer generative functions using any source (−0.51; 95% CI, −0.93 to −0.08). Other rural associations were not significant (Table S3).

## Discussion

Hospital concentration was associated with selected aspects of generative-AI use, while system affiliation and ACO participation were associated with all 5 outcomes. The unadjusted associations between high concentration, lower non–ML predictive use, and fewer generative functions weakened after adjustment. Hospital and community characteristics may therefore help explain these differences.

High concentration was associated with generative-AI use in both settings, while its association with any use was small and nonsignificant. Hospitals using only one setting are counted as users only in the alternative analysis. Hospitals may choose integrated and standalone tools for different tasks, depending on workflow needs and vendor offerings.^24^ This finding directs attention to how hospitals combine EHR-integrated and nonintegrated tools.

Function counts address a separate question: how many functions are supported by each source definition. The primary count requires both sources for the same function; the alternative includes functions using only one source. Moderate concentration was associated with fewer generative functions using both sources, while neither concentration category was significantly associated with the any-source count. The adjusted high-concentration estimate for the both-source count was also nonsignificant. The higher probability of generative-AI use and the lower function count therefore involved different concentration categories and different measures.

System affiliation and ACO participation were consistently associated with greater AI/ML use. Shared staff, infrastructure, purchasing, and oversight may support implementation, while ACO incentives may encourage prediction and care coordination.^5,6,21^ Everson and colleagues found lower current or planned EHR-integrated generative-AI use among independent hospitals in unadjusted analyses and greater use with participation in more alternative payment models after adjustment.^24^ Our findings extend this work to 2025 current use, predictive technologies, both EHR settings, and function counts, with a specific focus on ACO participation.

Related exchange studies also highlight vendor markets. Lin and colleagues examined hospital and EHR vendor concentration in relation to HRR exchange-network density.^25^ Everson and Adler-Milstein found that exchange engagement varied with vendor dominance and use of the dominant vendor.^26^ These studies support examining how vendor offerings and hospital resources together shape access to AI tools.

Rural hospitals had lower use for 2 outcomes: any non–ML predictive-model use and generative functions using any source. These measures include use in one EHR setting or through one tool source. The primary measures showed no significant rural–urban differences. The rural findings therefore depended on the definition of use. Staffing, capital, and implementation support are practical priorities for further research on these gaps.^1,22,23^

The consistent organizational associations and selected rural gaps support evaluating shared technical expertise and infrastructure for hospitals with fewer resources. Assistance could include staff training, tool evaluation, workflow integration, and ongoing monitoring, with community input into priorities and governance.^31^ Evaluations should assess access, implementation quality, and patient benefit.

This study has several limitations. Cross-sectional, self-reported data cannot establish causal effects. Implementation resources and decisions underlying tool use were not measured. Facility-level HHI may understate ownership concentration when commonly owned hospitals are treated separately. HRR-based HHI uses inpatient admissions and may not capture competition for outpatient, specialty, or digital services.^32^ It also does not measure EHR vendor concentration. Survey nonresponse and missing outcome or covariate data may introduce selection bias and limit generalizability. Retaining ACO nonrespondents reduced sample loss but cannot eliminate bias from nonrandom missingness. We did not compare retained and excluded hospitals by size, ownership, or region. Finally, some significant findings may reflect chance because we tested multiple outcomes and specifications without adjusting for multiple comparisons. Statistical significance under one definition but not another does not establish a statistically significant difference between the estimates.

## Conclusion

System affiliation and ACO participation were consistently associated with greater predictive-model and generative-AI use. Shared technical expertise, infrastructure, and implementation support merit evaluation as ways to help hospitals with fewer resources use these technologies effectively. Market concentration was not consistently associated with use across technologies and outcome definitions. Longitudinal studies are needed to assess whether concentration has cumulative effects on AI/ML use and whether AI/ML use, in turn, changes market concentration.

## Supplementary Material

qxag287_Supplementary_Data
