# Supplementary material for: Hospital market concentration and hospital adoption of artificial intelligence and machine learning
Source: Health Aff Sch. 2026 Oct 16;4(10):qxag287. doi: 10.1093/haschl/qxag287 (PMC13714907; doi:10.1093/haschl/qxag287)
Supplement: Supplementary file 1 [file qxag287_supplementary_data.zip › Supplement_1002.docx]

Supplementary material

Supplement 1. Outcome definitions and missing-response rules

Supplement 2. Descriptive results and analytic samples

Supplement 3. Sensitivity analyses

# Supplement 1. Outcome definitions and missing-response rules

## Current use by EHR integration setting

For ML-based predictive models, non-ML-based predictive models, and generative AI, current use is classified as:

Both: current use of EHR-integrated tools and tools that are not integrated into the EHR.

Only one: current use in either setting, but not both.
Neither: no current use in either setting.

**Primary analysis:** 1 = both; 0 = only one or neither.

**Alternative analysis:** 1 = either or both; 0 = neither.

## Function counts by tool source

The two tool sources are the EHR developer and another source (third-party or self-developed).

**Primary count:** each function = 1 if both sources are used for that function; 0 if only one or neither source is used.

**Alternative count:** each function = 1 if either or both sources are used; 0 if neither source is used.

Each function is counted at most once. Summing these indicators gives the predictive count (0–7) and generative count (0–8). Functions are listed in Table S2C.

## Missing-response rules

Planned use is not current use. If either response in an EHR-setting or tool-source pair is missing or “do not know,” the combined indicator is missing. Missing or unknown responses are not coded as nonuse. Primary and alternative analyses apply the same missing-response rules.

# Supplement 2. Descriptive results and analytic samples

## Table S2A. Current use by EHR integration setting

| **Use category** | **ML predictive N=1,959** | **Non-ML predictive N=1,817** | **Generative AI N=2,017** |
| --- | --- | --- | --- |
| Neither setting | 556 (28.4%) | 628 (34.6%) | 402 (19.9%) |
| Nonintegrated only | 140 (7.1%) | 131 (7.2%) | 211 (10.5%) |
| EHR-integrated only | 244 (12.5%) | 224 (12.3%) | 171 (8.5%) |
| Both settings | 1,019 (52.0%) | 834 (45.9%) | 1,233 (61.1%) |
| Any current use | 1,403 (71.6%) | 1,189 (65.4%) | 1,615 (80.1%) |

Values are numbers of hospitals (percentages). Denominators include hospitals with known responses for both EHR integration settings. The first four rows are mutually exclusive and sum to the denominator; percentages may not sum to 100 because of rounding. The primary outcome equals 1 for “Both settings” and 0 for the other three categories. “Any current use” combines both settings and the two only-one-setting categories; it is the sensitivity outcome, not a fifth mutually exclusive category. Source: 2025 AHA IT Supplement.

## Table S2B. Sample sizes

| **Outcome** | **Descriptive N** | **Regression N (all models)** | **Additional covariate exclusions** |
| --- | --- | --- | --- |
| ML predictive | 1,959 | 1,947 | 12 |
| Non-ML predictive | 1,817 | 1,804 | 13 |
| Generative AI | 2,017 | 2,004 | 13 |
| Predictive functions | 1,872 | 1,859 | 13 |
| Generative functions | 1,872 | 1,859 | 13 |

Descriptive samples include hospitals with classifiable outcome responses. Regression samples exclude hospitals missing other covariates; hospitals with missing ACO responses are retained as a separate category. The same outcome-specific regression sample is used for unadjusted, adjusted, primary, and alternative models.

## Table S2C. Primary function counts and indicators requiring both tool sources for each function

| **Measure (N=1,872 for each)** | **Mean** | **SD** |
| --- | --- | --- |
| Predictive function count (0–7) | 1.21 | 1.69 |
| Inpatient health trajectories or risks | 0.30 | 0.46 |
| High-risk outpatient identification | 0.23 | 0.42 |
| Health monitoring | 0.03 | 0.17 |
| Medical image interpretation | 0.12 | 0.32 |
| Treatment recommendations | 0.04 | 0.19 |
| Billing simplification or automation | 0.25 | 0.43 |
| Scheduling | 0.24 | 0.43 |
| Generative function count (0–8) | 0.54 | 0.98 |
| Patient-facing chatbot | 0.02 | 0.13 |
| Patient portal messaging | 0.06 | 0.23 |
| Ambient documentation | 0.07 | 0.25 |
| Discharge summaries | 0.02 | 0.14 |
| Nursing handoff summarization | 0.09 | 0.28 |
| Care-transition summarization | 0.07 | 0.25 |
| Prior authorizations | 0.09 | 0.28 |
| Billing code generation/suggestion | 0.14 | 0.35 |

Each function indicator equals 1 only when tools from both the EHR developer and another source are used for that same function, and 0 otherwise among known responses. Indicator means are proportions of hospitals meeting this definition (e.g., 0.30 = 30%). Count means are the average numbers of qualifying functions per hospital. Totals are computed before rounding, so rounded indicator means may not sum exactly to the rounded count mean. SD = standard deviation. Source: 2025 AHA IT Supplement.

# Supplement 3. Sensitivity analyses

## Any current use and function counts using any tool source

Sensitivity binary outcomes compare current use in one or both EHR integration settings with no current use in either setting. Sensitivity function counts include each function once if tools from either or both source categories are used. The models retain the primary analyses’ outcome-specific samples, covariates, missing-response rules, and HRR-clustered standard errors.

## Table S3. Full regression results for any current AI/ML use and function counts using any tool source

| **Covariate / contrast** | **ML predictive use (any setting) N=1,947** | **Non-ML predictive use (any setting) N=1,804** | **Generative AI use (any setting) N=2,004** | **Predictive functions (any source) N=1,859** | **Generative functions (any source) N=1,859** |
| --- | --- | --- | --- | --- | --- |
| **Model 1: Unadjusted** |  |  |  |  |  |
| Moderate vs low HHI | −2.9 (−11.7, 5.8) | 0.4 (−10.1, 11.0) | −3.9 (−11.1, 3.4) | −0.322 (−0.850, 0.207) | −0.309 (−0.808, 0.190) |
| High vs low HHI | −3.9 (−10.5, 2.8) | −5.7 (−14.2, 2.7) | −2.4 (−8.1, 3.2) | 0.068 (−0.378, 0.514) | −0.019 (−0.404, 0.366) |
| **Model 2: Adjusted** |  |  |  |  |  |
| Moderate vs low HHI | 3.3 (−2.7, 9.3) | 6.0 (−0.8, 12.8) | 0.4 (−5.3, 6.1) | 0.016 (−0.444, 0.475) | −0.105 (−0.580, 0.370) |
| High vs low HHI | 2.2 (−3.4, 7.9) | 0.2 (−6.8, 7.1) | 0.2 (−4.9, 5.2) | 0.120 (−0.237, 0.476) | 0.099 (−0.280, 0.478) |
| System affiliated vs independent | 35.4 (28.4, 42.4) | 48.9 (42.1, 55.7) | 22.0 (16.2, 27.8) | 1.701 (1.262, 2.140) | 1.266 (0.857, 1.675) |
| ACO participant vs nonparticipant | 23.1 (16.2, 30.0) | 19.6 (12.2, 27.0) | 17.9 (12.7, 23.1) | 1.445 (1.022, 1.867) | 0.671 (0.245, 1.097) |
| ACO nonresponse vs nonparticipant | 7.2 (−1.3, 15.7) | 7.2 (−2.6, 16.9) | 3.0 (−3.9, 9.8) | 0.315 (−0.178, 0.808) | −0.020 (−0.445, 0.406) |
| Rural vs urban | −5.2 (−10.8, 0.4) | −6.8 (−12.2, −1.4) | −4.2 (−9.7, 1.4) | −0.300 (−0.702, 0.103) | −0.507 (−0.930, −0.083) |
| Nongovernment vs government | 12.7 (5.5, 19.9) | 9.3 (1.4, 17.3) | 6.7 (1.2, 12.1) | 0.347 (−0.178, 0.872) | 0.153 (−0.375, 0.681) |
| <50 vs 50–99 beds | 3.4 (−3.1, 10.0) | 2.5 (−4.6, 9.6) | 4.6 (−1.7, 10.8) | 0.182 (−0.187, 0.551) | 0.466 (−0.012, 0.943) |
| ≥100 vs 50–99 beds | 9.2 (2.4, 16.0) | 7.1 (−0.5, 14.7) | 5.6 (−0.8, 12.0) | 0.134 (−0.236, 0.503) | −0.135 (−0.520, 0.250) |
| Teaching vs nonteaching | −0.3 (−4.7, 4.0) | −3.2 (−8.3, 2.0) | 1.1 (−3.1, 5.2) | 0.142 (−0.147, 0.432) | 0.045 (−0.246, 0.337) |
| RN staffing >75th percentile | 2.1 (−2.6, 6.7) | −2.2 (−7.5, 3.2) | 0.2 (−4.2, 4.6) | 0.075 (−0.208, 0.357) | −0.068 (−0.407, 0.270) |
| Medicare discharge rate >75th percentile | −4.9 (−9.7, −0.1) | −3.1 (−8.7, 2.4) | −5.4 (−9.9, −0.8) | 0.433 (0.171, 0.695) | 0.065 (−0.250, 0.381) |
| Medicaid discharge rate >75th percentile | 3.8 (−0.8, 8.3) | 5.7 (0.3, 11.1) | 2.6 (−2.3, 7.5) | 0.346 (0.064, 0.628) | 0.350 (0.004, 0.696) |
| County FQHC supply >75th percentile | −3.1 (−8.1, 1.9) | 0.4 (−5.0, 5.7) | −2.5 (−7.0, 2.0) | −0.275 (−0.561, 0.011) | −0.100 (−0.399, 0.199) |
| County minority-race share >75th percentile | −1.1 (−6.0, 3.8) | 1.7 (−4.2, 7.7) | −8.1 (−12.4, −3.9) | −0.251 (−0.580, 0.079) | 0.074 (−0.259, 0.407) |
| County age ≥65 share >75th percentile | −0.4 (−5.8, 4.9) | 4.9 (−1.0, 10.8) | 2.6 (−2.2, 7.4) | −0.372 (−0.762, 0.019) | 0.002 (−0.370, 0.374) |
| County median income >75th percentile | 0.8 (−4.6, 6.2) | −1.1 (−6.6, 4.3) | 1.2 (−3.0, 5.4) | 0.008 (−0.263, 0.279) | 0.174 (−0.107, 0.455) |

Notes: Values are average marginal effects (95% confidence intervals). Binary outcomes are percentage-point differences; count outcomes are differences in numbers of functions. Model 1 includes HHI only; Model 2 includes all covariates shown. Logistic regressions were used for binary outcomes and Poisson regressions for counts. Standard errors are clustered by HRR. Both models use identical outcome-specific samples. Confidence intervals are not adjusted for multiple comparisons.

Reference categories are shown in each contrast. For percentile-based covariates, the reference is the 75th percentile or below. RN staffing is full-time-equivalent registered nurses per 1,000 inpatient days. FQHC denotes federally qualified health center; county racial composition is the share identifying as a minority race. ACO nonresponse is a separate category. HHI: low <1,000; moderate 1,000–1,800; high >1,800.

Binary outcomes equal 1 for current use in one or both EHR integration settings and 0 for use in neither setting. Each function counts once if tools from the EHR developer, another source, or both are used; functions with neither source used count as 0 among known responses. Count ranges are 0–7 predictive and 0–8 generative. AI = artificial intelligence; ML = machine learning; ACO = accountable care organization; EHR = electronic health record; HRR = hospital referral region; HHI = Herfindahl-Hirschman Index.
